# Ferroptosis: a novel pathogenesis and therapeutic strategies for Parkinson disease: A review

**DOI:** 10.1097/MD.0000000000041218

**Published:** 2025-01-17

**Authors:** Di Jiao, Yang Yang, Kejing Wang, Yaomei Wang

**Affiliations:** a School of Medicine, Zhengzhou University of Industrial Technology, Zhengzhou, China; b Department of Hematology, Henan Cancer Hospital, the Affiliated Cancer Hospital of Zhengzhou University, Zhengzhou, China.

**Keywords:** ferroptosis, Parkinson disease, pathogenesis, therapeutic strategies

## Abstract

Parkinson disease (PD) is the second most common neurodegenerative disease, and its incidence is climbing every year, but there is still a lack of effective clinical treatments. In recent years, many studies have shown that ferroptosis plays a key role in the progression of PD. Most importantly, many cellular and animal studies and clinical trials have shown that episodes of PD can be alleviated by inhibiting the ferroptosis process, such as utilizing inhibitors, chelating agents, and others. Here, we review the role of ferroptosis, a new form of cell death, in the pathogenesis of PD, and summarize the therapeutic strategies for targeting ferroptosis in PD, hoping to provide new thinking for the study of PD pathogenesis and the development of therapeutic strategies.

## 1. Introduction

Parkinson disease (PD) is a neurodegenerative disease mainly caused by the loss of dopaminergic neurons in the compact part of the substantia nigra (SN) and abnormal aggregation of alpha-synuclein (α-syn). PD pathogenesis is complex, with multiple molecular pathways associated with its pathology, including oxidative stress, inflammatory response, abnormal protein metabolism, ferroptosis, and so on.^[1]^ Among them, ferroptosis, as a newly recognized form of cell death in dopaminergic neurons in PD, has recently been extensively and intensively studied. Currently, there is no cure for PD, and existing treatments, mainly anticholinergic drugs (e.g., phenazopyridine) and dopaminergic drugs (e.g., levodopa), are insufficient to meet the needs of the patients. Ferroptosis may be a promising strategy to improve the treatment of PD.^[2]^ Here, we systematically review the molecular mechanism of ferroptosis and its research progress in PD, combining the research results and current status. In addition, therapeutic strategies targeting ferroptosis in PD are also discussed, and we believe that these findings will help develop more potential targets and pharmacological small molecules in future PD therapy to mitigate or even cure the onset of PD. SANRA details are added in Table S1, Supplemental Digital Content,^[3]^ http://links.lww.com/MD/O263.

## 2. Molecular mechanisms of ferroptosis

**Figure 1.**
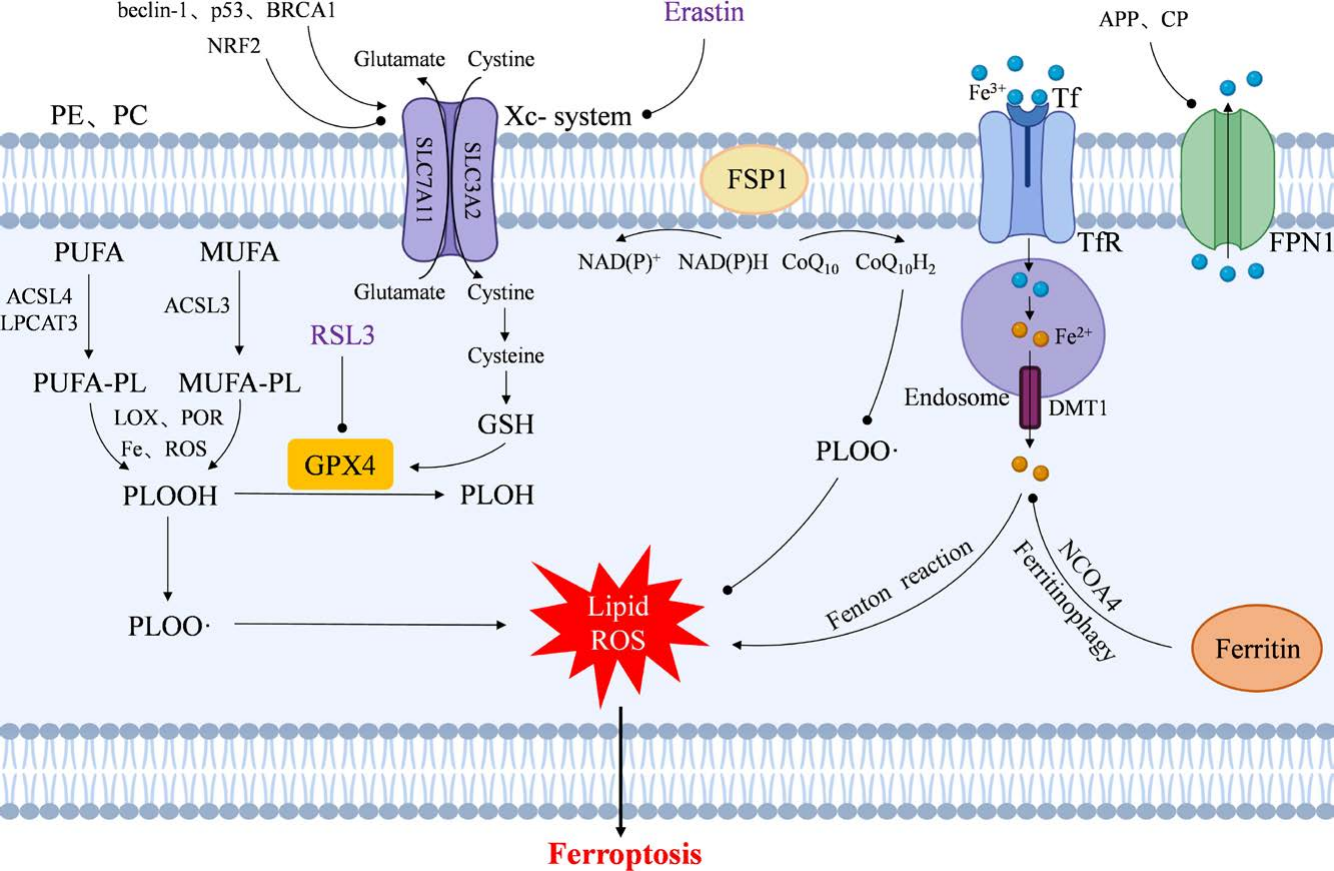
Major molecular mechanisms of ferroptosis.

Ferroptosis is a regulated ferroptosis-dependent cell death characterized by lipid peroxidation and iron overload, which is closely associated with a variety of biological processes, including lipid peroxidation, dysregulation of antioxidant defenses, iron accumulation, and a variety of disease-related signaling pathways.^[4]^ Ferroptosis occurs due to the accumulation of iron-dependent lipid peroxides produced by reactive oxygen species (ROS),^[5]^ the molecular mechanisms of which are mainly involved in the following (Fig. 1).

### 2.1. Lipid peroxidation

The initiation of ferroptosis is closely related to the accumulation of lipid peroxides in cell membranes, and among a wide range of membrane phospholipids, phosphatidylcholine (PC) and phosphatidylethanolamine (PE) are the main targets of lipid peroxidation.^[6]^ Free polyunsaturated fatty acid (PUFA) is first catalyzed by acyl-coenzyme A synthetase long-chain family member 4 (ACSL4), lysophosphatidylcholine acyltransferase 3 (LPCAT3) to form PUFA-containing phospholipids (PUFA-PL). Next, under iron-rich and ROS-rich conditions, peroxidation occurs via lipoxygenase (LOX), cytochrome P450 oxidoreductase (POR), etc, resulting in the formation of phospholipid peroxides (PLOOH), which lead to ferroptosis by either disrupting cellular membranes or generating lipid-derived electrophilic small molecules.^[7]^ Monounsaturated fatty acids (MUFA) are also involved in ferroptosis via ACSL3, but MUFA significantly reduced the oxidative susceptibility of membrane lipids.

Yang et al found that pretreatment of cells with D-PUFA containing the heavy hydrogen isotope deuterium at the bis-allyl site effectively prevented PUFA oxidation and blocked ferroptosis. Meanwhile, the use of RNAi and inhibitors to reduce LOX levels effectively alleviated ferroptosis. Phosphorylase kinase G2 (PHKG2) has also been found to be involved in ferroptosis by regulating intracellular iron content, affecting LOX activity, and controlling the oxidative reaction of PUFA.^[8]^

### 2.2. Dysregulation of the antioxidant defense system

Dysregulation of antioxidant defenses, especially Xc^-^-GSH-GPX4-dependent dysregulation of antioxidant defenses, is an important factor in the induction of cellular ferroptosis. Lipid peroxides (PLOOH) are key intermediates in the process of lipid peroxidation and can be converted to lipid alcohols (PLOH) by glutathione peroxidase 4 (GPX4). Normal GPX4 activity is essential for maintaining lipid membrane homeostasis, preventing free radical formation and accumulation of toxic lipid peroxides, and thus reducing ferroptosis.^[9]^ Glutathione (GSH) acts as a cofactor for GPX4, and its depletion can lead to inactivation of GPX4. GSH synthesis is regulated by cysteine and the Xc^-^ amino acid transporter system on the cell membrane transports extracellular cystine into the cell for further reduction to cysteine. As the most commonly used ferroptosis inducers, erastin and RSL3 induce ferroptosis by targeting the Xc^-^-GSH-GPX4 system. Erastin targets cystine uptake by the Xc^-^ system inhibits GSH synthesis, and reduces the level of cellular antioxidant defenses; it also binds to Voltage-dependent anion channel (VDAC2/VDAC3), leading to an increase in mitochondrial membrane permeability and accelerated oxidation, resulting in ROS accumulation. 1S,3R-RSL3 (RSL3), on the other hand, directly inactivates GPX4 and induces a lethal accumulation of lipid peroxides, leading to ferroptosis.

In addition, ferroptosis suppressor protein 1 (FSP1) can catalyze the regeneration of lipophilic antioxidant coenzyme Q10 (coenzyme Q10, CoQ10) via NAD(P)H, which traps lipid peroxidation free radicals in a GPX4-independent form, hindering lipid peroxidation and inhibiting iron death.^[10]^ The FSP1-NAD(P)H-CoQ10 pathway synergizes with the Xc^-^-GSH-GPX4 system for antioxidant defense and suppresses ferroptosis.^[11]^

### 2.3. Iron accumulation

When iron levels are imbalanced, elevated unstable iron ions lead to cellular damage by catalyzing the production of toxic free radicals, especially through the fenton reaction, mainly hydroxyl radicals (HO) produced by the activation of hydrogen peroxide (H_2_O_2_) by ferrous ions (Fe^2+^), which possesses strong oxidative capacity and leads to ferroptosis.^[12]^ Thus, iron metabolism may influence cellular sensitivity to ferroptosis. The up-regulation of transferrin receptor (TfR) expression and the down-regulation of ferritin heavy chain 1 (FTH1) and ferritin light chain (FTL) expression in ferroptosis-sensitive cells (e.g., BJeLR cells) further suggests that iron metabolism affects ferroptosis. Iron levels depend on the coordination between iron input, output, storage, and turnover. Fe^3+^ is transported from the extracellular milieu to endosomes via transferrin (Tf) and TfR, reduced to Fe^2+^, and later transported to the cytoplasm via divalent metal transporter protein 1 (DMT1) while being regulated by iron-responsive element-binding protein 1/2 (Iron-responsive element-binding protein 1/2) at the messenger RNA level.^[13]^ Iron export, on the other hand, is mainly dependent on membrane iron transport protein 1 (ferroportin 1, FPN1) and is regulated by β-amyloid precursor protein (APP) and ceruloplasmin (CP).^[14,15]^ In addition, autophagic degradation of cellular iron storage proteins mediated by nuclear receptor coactivator 4 (NCOA4), known as ferritinophagy, can further influence cellular ferroptosis susceptibility by affecting iron metabolism.^[16]^ Other proteins that affect cellular iron metabolism, such as CDGSH iron sulfur domain 1 and heat shock protein family B member 1 (HSPB1), also affect ferroptosis sensitivity.^[17,18]^

### 2.4. Systemic regulation

The process of ferroptosis is subject to systematic regulatory effects of multiple genes. The Xc^-^ amino acid transport system is structurally composed of Solute carrier family 7 member 11 (SLC7A11) and SLC3A2, and overexpression of SLC7A11 by gene transfection reduced erastin-induced ferroptosis. The autophagy regulator beclin-1 inhibits Xc^-^ system activity and hinders cysteine uptake through interaction with SLC7A11 to induce ferroptosis.^[19]^ Activation of tumor suppressor p53 and Breast cancer 1 -associated protein 1 participates in ferroptosis by repressing SLC7A11 transcription.^[19–21]^ Nuclear factor erythroid 2-related factor 2 (NRF2) upregulated SLC7A11 expression, while directly or indirectly regulating GPX4 expression to protect cells from ferroptosis by promoting the antioxidant pathway and limiting ROS accumulation; whereas, knockdown of NRF2 and NRF2 target genes accelerated erastin or sorafenib-induced ferroptosis.^[22]^ Meanwhile, the knockdown of GPX4 led to lipid peroxidation and induced ferroptosis in an iron, mitogen-activated protein kinase kinase (MEK) and ROS-dependent manner, whereas overexpression of GPX4 produced resistance to RSL3.^[23]^ The typical Nicotinamide adenine dinucleotide phosphate oxidase (NOX) inhibitor diphenyleneiodonium and the NOX1/4-specific inhibitor GKT137831 were also found to partially inhibit erastin-induced ferroptosis in Calu-1 and HT1080 cells. Knockdown of cysteinyl-tRNA synthetase (CARS) inhibits ferroptosis induced by erastin, whereas overexpression of CARS enhances the sensitivity of several cancer cells to erastin.^[24]^ Overexpression of HSPB1 was also found to inhibit erastin-induced ferroptosis.^[25]^ Furthermore, the knockdown of the lysosomal protein prosaposin mediates neuronal ferroptosis by triggering lipofuscin formation.^[26]^

## 3. Ferroptosis in Parkinson disease

**Figure 2.**
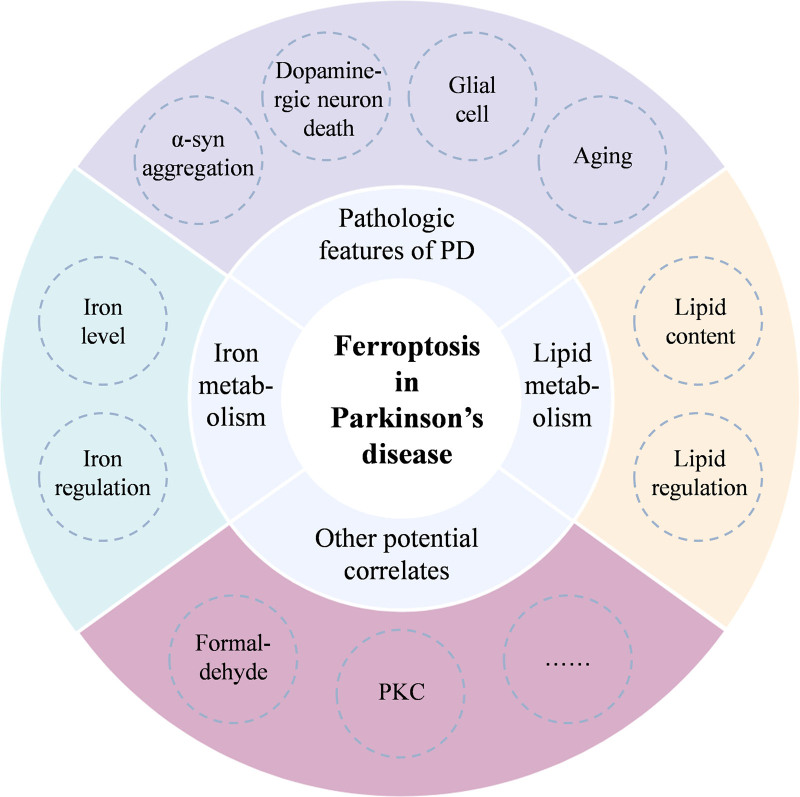
Factors influencing ferroptosis in Parkinson disease.

Many neurodegenerative disease pathologies are associated with ferroptosis, and as early as 1987, altered PUFA composition and increased lipid peroxidation and ferroptosis in SN cells of dead PD patients were found.^[27]^ Biondetti et al found that increased iron levels were associated with neuronal loss in the compact part of SN and striatal dysfunction.^[28]^ In 1-methyl-4-phenyl-1,2,3,6-tetrahydropyridine (MPTP), rotenone, paraquat (PQ), 6-hydroxydopamine (6-OHDA) induced PD models, ferroptosis has been proved to be the main form of cell death.^[29]^ Aggregation of the PD pathologic marker protein α-syn has also been found to be associated with iron, calcium, and lipid peroxidation.^[30]^ These features suggest a potential link between ferroptosis and PD. Therefore, investigating the molecular mechanisms of ferroptosis may reveal potential targets for controlling the progression of PD and performing PD therapy. We discuss the correlation between PD pathogenesis and ferroptosis in the following section (Fig. 2).

### 3.1. Susceptibility to ferroptosis due to pathologic features of Parkinson disease

α-syn is 1 of the important features of PD, and a growing number of studies have shown that its aggregation generates ROS that accelerate lipid peroxidation and induce ferroptosis. In the human stem cell-derived synucleinopathy model constructed by Angelova et al, intracellular α-syn aggregates bind to membranes and induce ferroptosis through their interaction.^[30]^ Meanwhile, ferroptosis-inhibiting drugs, such as D-PUFA and iron chelators, have been shown to prevent neuronal cell death triggered by toxic α-syn oligomers, highlighting a potential connection between α-syn and ferroptosis.^[30]^ Several researchers have found that decreased α-syn expression in human dopaminergic neurons prevents ferroptosis, whereas elevated α-syn expression of SNCA triploid patient origin leads to increased susceptibility to lipid peroxidation and ferroptosis.^[31]^ In some PD cells and mouse models, α-syn accumulation was found to decrease the NRF2 target genes aldo-keto reductase family 1 member C1 and glutamate cysteine ligase modulating subunit expression, by inhibiting the NRF2 protein level, thereby increasing the sensitivity to ferroptosis.^[32]^

Another feature of PD is the death of dopaminergic neurons in the compact part of SN, followed by a decrease in dopamine (DA) levels. DA is a strong inhibitor of ferroptosis, and the loss of DA in PD increases cellular vulnerability to ferroptosis. It’s been proven that DA inhibits iron-mediated ROS production by increasing the stability of GPX4.^[33]^

Glial cells play a key role in the inflammatory response to PD, and several studies have shown that glial cells are also involved in regulating ferroptosis processes. Oligodendrocytes have a high iron content among central nervous system cells, and ferritin-driven rapid iron release promotes lipid peroxidation and cell death, which leads to dopaminergic neuron ferroptosis.^[34]^ Ishii et al found that astrocytes deliver relevant transcripts and proteins to help protect neurons from oxidative stress and thus ferroptosis.^[35]^ Furthermore, inflammatory signals upregulated inducible nitric oxide synthase (iNOS) in microglial cells, decreasing 15-LOX activity and thus inhibiting ferroptosis.^[36]^

Much evidence also suggests that aging may be a potential cue linking PD and ferroptosis. Elevated expression of heme oxygenase-1 (HO-1) in human astrocytes has been reported to contribute to PD features, and is also associated with Fe^3+^ deposition.^[37]^ Cristina et al found higher levels of HO-1 expression and greater alterations in iron metabolism-related proteins in microglial cells of older mice (15–18 months) compared to younger mice (3–4 months). Age-related increases in HO-1 expression affect oxidative stress, iron metabolism, and inflammation, eventually leading to ferroptosis, whereas suppressing HO-1 expression mitigates these effects.^[38]^

### 3.2. Abnormalities of lipid metabolism in Parkinson disease and ferroptosis

Dysregulated lipid metabolism represents a shared pathological characteristic of both PD and ferroptosis. PUFA phospholipids are the target of lipid peroxidation, this process can destroy the phospholipid structure of bilayer cell membranes, which is 1 of the major causes of ferroptosis. Lipid changes are observed in PD patients and healthy populations, and lipids are involved in metabolic pathways regulated by PD risk genes.^[39]^ In addition, 4-hydroxy-2-nonenal (HNE), a toxic end product of free radical-stimulated peroxidation, has increased mean concentrations in the cerebrospinal fluid and SN of patients with PD, and high levels of HNE can alter DA transport pathways and lead to DA loss.^[40]^

In addition, stress stimulates the release of corticosteroids, activates the protein kinase C (PKC) pathway, and induces the interaction of 15-LOX with phosphatidylethanolamine binding protein 1 (PEBP1), resulting in the formation of 15-HpETE-PE complex, which promotes PD progression by triggering plasma membrane phospholipid peroxidation leading to ferroptosis in dopaminergic neurons.^[41]^ Ca^2+^-independent phospholipase A2β (iPLA2β) has been found to act as an important regulator of ferroptosis, allowing cells to avoid ferroptosis by hydrolyzing the 15-HpETE-PE complex. iPLA2β expression is reduced in PD, leading to the development of PD.^[42]^ Overexpression of thioredoxin 1 has also been found to inhibit ferroptosis and ameliorate behavioral deficits in PD mice by inhibiting the reduction of GPX4 and GSH and the increase of ROS.

### 3.3. Abnormalities of iron metabolism in Parkinson disease and ferroptosis

Abnormalities of iron metabolism act as a bridge between PD and ferroptosis.^[43]^ PD is characterized by neuronal loss, along with alterations in iron accumulation and distribution, and tissue atrophy and changes in iron content are important markers of brain aging.^[44]^ Several studies have shown that SN iron levels are also associated with PD dyskinesia and degeneration of dopaminergic neurons.^[45,46]^ In addition, plasma ferritin levels and iron deposition in the deep gray matter nuclei showed an inverse correlation with the severity and duration of PD.^[47]^ Bergsland et al found that iron levels in the posterior part of the ventral SN were associated with PD progression by quantitative susceptibility mapping analysis.^[48]^

In various PD animal and cellular models and brain tissues of PD patients, iron regulation-related protein expression is dysregulated, and iron, Tf, and TfR2 levels are high in dopaminergic neurons.^[49]^ It was found that overexpression of TfR2 resulted in the uptake and targeting of extracellular Tf-bound iron to mitochondria, while TfR2 deficiency was shown to inhibit iron overload and degeneration of dopaminergic neurons.^[50]^ Therefore, excessive iron accumulation in mitochondria via the Tf/TfR2 system may be a critical factor in the progression of PD. Several studies have shown that reduced levels of APP and CP in PD patients lead to a decreased ability of FPN1 to export iron resulting in iron accumulation and oxidative stress production in the SN.^[51,52]^ Transferrin was also found to significantly ameliorate motor deficits in the MPTP-induced PD mouse model by down-regulating DMT1 and ACSL4 and up-regulating FSP1, acting as a neuroprotective effect.^[53]^

### 3.4. Other potential correlates of ferroptosis in Parkinson disease

Formaldehyde, a common cause of PD, reduces the expression of tyrosine hydroxylase, a key enzyme in DA synthesis, and induces PD-like neuronal damage.^[54]^ In addition, formaldehyde increases intracellular iron concentration and upregulates ferroptosis-related genes, such as prostaglandin-endoperoxide synthase 2, glutaminase 2, SLC1A5, and SLC38A1 to induce ferroptosis in hippocampal neurons.^[55]^ This suggests that formaldehyde may play a key role in ferroptosis in PD. Furthermore, in a model of PD in LUHMES cells, erastin-induced ferroptosis was triggered by PKC activation to activate MEK in a RAS-independent manner.^[56]^ More potential correlates of ferroptosis still urgently need to be explored.

## 4. Therapeutic strategies to target ferroptosis in Parkinson disease

### 4.1. Modulators targeting lipid peroxidation

Lipid peroxidation is a key process in ferroptosis; therefore, lipid peroxidation inhibitors and antioxidants have been widely investigated as important means to inhibit ferroptosis and thus mitigate the development of PD (Table 1).

**Table 1:** Modulators targeting ferroptosis in Parkinson disease.

	Form	Modulator	Action site
Targeting lipid peroxidation	Lipid peroxidation inhibitor	BHB	ACSL4
		CuATSM	Lipid peroxidation and free radical
		Leonurine	15-LOX/PEBP1
	Antioxidant	Fer-1	GPX4, SNX5
		NAC	GSH, DA transporter
		Lapatinib	GPX4/GSH/NRF2, ACSL4
		Selenium	TFAP2c, Sp1→GPX4
		Vitamin E + omega-3 fatty acids	GSH
		CoQ10	Free radical
		Idebenone	NAD(P)H dehydrogenase (quinone) 1, GPX
		Resveratrol	NFR2/Keap1/SLC7A11
		GSH	GSH
Targeting iron accumulation	Iron chelator	CQ	Fe, ROS
		ALA	SIRT1/NRF2
		DFO	DMT1, TfR1, GPX4, FTH1, ACSL4
		DFP	Fe, α-syn
		1-hydroxypyrazin-2(1H)-1	ROS
	Ferritinophagy inducers	Rapamycin	Ferritinophagy

Abbreviations: ACSL4 = acyl-coenzyme A synthetase long-chain family member 4, ALA = alpha-lipoic acid, BHB = beta-hydroxybutyrate, CQ = clioquinol, CoQ10 = coenzyme Q10, CuATSM = copper compound Copper(II)-diacetyl-bis(N4-methylthiosemicarbazone), DA = dopamine, DFO = desferrioxamine, DFP = deferiprone, DMT1 = divalent metal transporter protein 1, Fer-1 = ferrostatin-1, FTH1 = ferritin heavy chain 1, GPX4 = glutathione peroxidase 4, GSH = glutathione, LOX = lipoxygenase, NAC = N-acetyl cysteine, NADPH = Nicotinamide adenine dinucleotide phosphate, NRF2 = nuclear factor erythroid 2-related factor 2, PEBP1 = phosphatidylethanolamine binding protein 1, SIRT1 = Sirtuin 1, SLC7A11 = solute carrier family 7 member 11, SNX = sorting nexin, Sp1 = specific protein 1, TFAP2c = transcription factor activating enhancer binding protein 2 gamma, TfR1 = transferrin receptor 1, ROS = reactive oxygen species.

Beta-hydroxybutyrate (BHB), 1 of the major components of ketone bodies, reduces lipid peroxidation by down-regulating ACSL4 via zinc finger protein 36, thereby attenuating ferroptosis and dopaminergic neuronal damage in the PD model.^[57]^ Copper compound copper(II)-diacetyl-bis(N4-methylthiosemicarbazone) (CuATSM) inhibits ferroptosis in the PD model by blocking lipid peroxidation and free radical production, and maybe a clinical candidate for the treatment of PD.^[58]^ PKC inhibitors are also strong candidates for pharmacological modulation of ferroptosis signaling cascades, and leonurine, a natural product isolated from herbaceous plants, attenuates membrane phospholipid peroxidation activity by inhibiting the 15-LOX/PEBP1 interaction.^[41]^ They have been found to alleviate the pathological process of PD as lipid peroxidation inhibitors.

Also, a variety of antioxidants have been found to influence PD pathology by inhibiting ferroptosis. Ferrostatin-1 (Fer-1) is a well-known ferroptosis inhibitor that neutralizes alkoxyl radicals, mitigates excessive iron accumulation, and eliminates lipid peroxides.^[59]^ Huang et al found that Fer-1 inhibited ferroptosis in 6-OHDA-induced PD rats and cellular models.^[60]^ Meanwhile, Fer-1 significantly ameliorated PD-associated motor behavior and pathological changes in multiple in vivo and in vitro PD models.^[61]^ The results of several clinical trials have shown that high-dose intravenous injection of N-acetyl cysteine (NAC), a cysteine precursor, increased blood GSH levels and increased DA transporter binding in the caudate and chitin nuclei of PD patients and healthy controls.^[62,63]^ In a clinical trial of 42 patients with PD, participants were randomized into 2 groups; the treatment group received 50 mg/kg NAC intravenously weekly plus 500 mg NAC orally twice a day for 3 months, and the control group received only standard treatment. Dopamine transporter (DAT) binding was measured by DaTscan. Compared with the control group, patients in the NAC group showed a significant increase in DAT binding in the caudate and shell nuclei (3.4% –8.3%) and a significant improvement in PD symptoms. In another clinical trial in 3 patients with PD and 3 healthy controls, a single injection of 150 mg/kg NAC was used to determine brain GSH concentration by 7-T magnetic resonance spectroscopy and to determine the GSH redox ratio in the blood of each subject. The results showed that NAC increased the blood GSH redox ratio and brain GSH concentration in PD patients and healthy controls. Lapatinib, an anticancer drug, activates the GPX4/GSH/NRF2 pathway, inhibits ACSL4, and enhances antioxidant capacity, thereby alleviating iron-dependent ferroptosis and ameliorating PD symptoms.^[64]^ Selenium enhances GPX4 expression through synergistic activation of the transcription factors specific protein 1 and transcription factor activating enhancer binding protein 2 gamma, which in turn inhibits ferroptosis and reduces motor retardation and DNA damage in the PQ-induced PD rat model.^[65,66]^ A randomized, double-blind, placebo-controlled clinical trial of 60 patients with PD conducted by Taghizadeh et al confirmed that 1000 mg of vitamin E in combination with 400 IU of omega-3 fatty acids for 12 weeks increased GSH concentrations and significantly improved symptoms in patients with PD.^[67]^ CoQ10, an important component of the FSP1 pathway, traps free radicals and reduces lipid peroxidation levels, and has been observed in clinical trials to significantly improve PD symptoms when administered orally at 300 mg/day for 48 weeks.^[68]^ Idebenone is a CoQ10 analog with high antioxidant capacity, inhibits the down-regulation of NAD(P)H dehydrogenase (quinone) 1 expression, reduces lipid peroxidation levels in the striatum, increases GPX4 expression, regulates ferroptosis, and attenuates locomotor deficits and TH loss in a rat model of PD, and exerts neuroprotective effects.^[69]^ Resveratrol inhibits free iron production and attenuates cellular inflammation and oxidative stress through the NFR2/Keap1/SLC7A11 pathway in the rotenone-induced PD cell model.^[70]^ GSH may also serve as a potential agent for the treatment of PD, however, its efficacy varied across 2 clinical studies. Hauser et al observed no improvement in PD symptoms with 1400 mg of GSH administered intravenously 3 times a week for 4 weeks compared to placebo, while Mischley et al observed improved motor function in PD patients with intranasal intake of 100/200 mg GSH 3 times a day for 3 months.^[71,72]^

### 4.2. Modulators of targeted iron accumulation

Iron homeostasis affects cellular sensitivity to iron death, which in turn affects PD-associated pathophysiological pathways, and targeted therapies against iron accumulation, such as iron chelators and ferritinophagy inducers, may be potent candidates to pharmacologically modulate the iron signaling cascade and regulate the PD pathological process through the ferroptosis pathway (Table 1). Shi et al found in the PD monkey model that clioquinol (CQ) treatment reduced ROS levels and iron accumulation in the SN, while significantly improving dyskinesia and nonkinesia in this model.^[73]^ The natural compound alpha-lipoic acid acts as an antioxidant and iron chelator to regulate iron metabolism and inhibit iron-dependent ferroptosis via Sirtuin 1/NRF2 pathway signaling, thereby improving PD symptoms.^[74]^ The iron chelator desferrioxamine (DFO) inhibits ferroptosis and protects neurons through DMT1, TfR1, GPX4, FTH1, and ACSL4 in the PD cell model.^[75]^ Deferiprone (DFP), another iron chelator, was found to reduce iron deposition, inhibit α-syn pathotoxicity in SN, and alleviate dyskinesia in the PD animal model.^[76]^ In several clinical trials, an early study by Devos et al found a reduction in iron accumulation in SN and an increase in exercise UPDRS scores in patients with early PD treated with DFP (30 mg/kg/day) for 12 months.^[77]^ However, a recent phase II placebo-controlled trial they recently conducted showed that patients treated with DFP had more severe symptoms after 36 weeks than those treated with placebo.^[78]^ Therefore, larger and more rigorous clinical trials are needed in the future to further determine the therapeutic effect of DFP on PD. A novel iron chelator 1-hydroxypyrazine-2(1H)-1 synthesized by Lewis et al was found to delay progression in the PD cell model by blocking ROS production.^[79]^ In addition, rapamycin, an autophagy inducer, has been found to affect iron metabolism through the activation of ferritinophagy and to inhibit iron-dependent ferroptosis, thereby improving PD characteristics.^[80]^

Although many ferroptosis inhibitors are continually found to target PD pathologic features, most of them remain difficult to use successfully in the clinic. Some drugs have inhibitory effects on ferroptosis, but poor stability, low solubility, low targeting, low safety, and poor pharmacokinetics limit their safety and widespread use, and therefore have not entered clinical studies.^[81,82]^ The drugs entering clinical studies still lack long-term clinical data, while some toxic side effects (e.g., nephrotoxicity, etc) limit their scope of application and potential for clinical use. Such as in a 12-month phase II clinical trial, DFP significantly improved motor function in patients with PD, but with continued DFP, their improvement in motor performance was diminished and showed neutropenia.^[76]^ CuATSM was neuroprotective in phase I clinical trials of ALS and PD, however, in phase II clinical trials of ALS, patients were found to have reduced microglia and immune function.^[83]^ Therefore, researchers need to further optimize drugs and develop selective ferroptosis inhibitors for targeted drug delivery, improved drug utilization, and reduced drug side effects.

## 5. Conclusion

In recent years, ferroptosis has attracted much attention in neurodegenerative disease research because of its important role in the progression of PD pathogenesis. Multiple lines of evidence suggest that a variety of risk factors for ferroptosis, including abnormalities of lipid metabolism and abnormalities of iron metabolism, are associated with PD pathology. In addition, some PD pathologic features are involved in the process of ferroptosis.^[84]^ Therefore, an intricate and close relationship between PD and ferroptosis is believed to exist, making the ferroptosis pathway a promising target for PD therapy. Several therapeutic strategies targeting ferroptosis in PD have been identified after extensive research on them, including lipid peroxidation inhibitors, antioxidants, iron chelators, ferritinophagy activators, etc, which have demonstrated excellent anti-ferroptosis and anti-PD effects in the PD model and clinical trials.

In addition, targeted modulation of ferroptosis in combination with existing PD therapies offers a promising avenue to delay progression and mitigate the side effects of existing treatments. For example, L-DOPA is often given to PD patients in the clinic, and although its long-term use can effectively supplement the dopamine deficiency, adverse effects such as dyskinesia and psychiatric symptoms may occur. The selection of appropriate ferroptosis inhibitors for combined use may be able to alleviate the L-DOPA-induced side effects and improve the long-term quality of life of patients.^[85]^ In addition, ferroptosis inhibitors reduce inflammation and neuronal damage by attenuating neuronal injury from lipid peroxidation and iron accumulation. Combining strategies targeting ferroptosis with existing PD therapies, which act synergistically through multiple mechanisms, helps to address the pathologic features of PD while improving the efficacy and safety of existing treatments. Therefore, there is a need to further explore PD treatments incorporating ferroptosis, which will provide new opportunities for our future research directions.

Ferroptosis is an inevitable consequence of PD and a fundamental factor in the development and progression of PD. Modulating ferroptosis in PD demonstrates promising neuroprotective effects, offering a novel avenue for therapeutic intervention. Therefore, continuing to explore the relationship between PD and ferroptosis will help to identify new PD therapeutic targets and treatment strategies, and motivate researchers to develop more ferroptosis inhibitors for future PD treatment.

## Author contributions

**Data curation:** Di Jiao, Yang Yang, Kejing Wang.

**Writing – original draft:** Di Jiao.

**Writing – review & editing:** Yaomei Wang.

## Supplementary Material


