# Supplementary material for: Ferroptosis: a novel pathogenesis and therapeutic strategies for Parkinson disease: A review
Source: Medicine (Baltimore). 2025 Jan 17;104(3):e41218. doi: 10.1097/MD.0000000000041218 (PMC11749581; doi:10.1097/MD.0000000000041218)
Supplement: Supplementary file 1 [file medi-104-e41218-s001.docx]

Supplementary table 1: Scale for the Assessment of Narrative Review Articles (SANRA) details

| Scale for the Assessment of Narrative Review Articles (SANRA) | |
| --- | --- |
| 1. Justification of the article’s importance for the readership | In recent years, a large number of studies have shown that ferroptosis plays a key role in PD progression, but there are fewer reviews related to the association between the two and the therapeutic strategy of PD from the perspective of ferroptosis, so this review hopes to provide some help for researchers. |
| 2. Statement of concrete aims or formulation of questions | Here, we review the role of ferroptosis, a new form of cell death, in the pathogenesis of PD, and summarize the therapeutic strategies for targeting ferroptosis in PD, hoping to provide new thinking for the study of PD pathogenesis and the development of therapeutic strategies. |
| 3. Description of the literature search | The literature for this review was mainly obtained from Pubmed, and the search terms included “parkinson's disease”, “ferroptosis”, “molecular mechanisms”, “treatment”, and logical expressions such as “Parkinson's disease and iron death”, “Parkinson's disease and iron death and molecular mechanisms” etc. to further specify the search target. |
| 4. Referencing | Key sections of the review are supported by appropriate literature. |
| 5. Scientific reasoning | The review systematically summarizes and analyzes the content of the relevant references and provides clear and logical arguments. |
| 6. Appropriate presentation of data | The relevant data in the article are clearly documented. |
